# The broad‐spectrum antibiofilm activity of amyloid‐forming hexapeptides

**DOI:** 10.1111/1751-7915.13721

**Published:** 2020-11-28

**Authors:** Dongru Chen, Jing Li, Ting Pan, Ruixue Wu, Ye Tao, Huancai Lin

**Affiliations:** ^1^ Guangdong Provincial Key Laboratory of Stomatology Department of Orthodontics Hospital of Stomatology Guanghua School of Stomatology Sun Yat‐sen University Guangzhou Guangdong China; ^2^ Guangdong Provincial Key Laboratory of Stomatology Department of Preventive Dentistry Hospital of Stomatology Guanghua School of Stomatology Sun Yat‐sen University Guangzhou Guangdong China

## Abstract

Evidence suggests that short amyloid‐forming peptides derived from bacterial proteomes have functional roles; however, the reported activities are diverse and the underlying mechanisms remain unclear. In this study, we simulated short amyloid‐forming peptides from the amyloid‐forming truncated protein C123 of *Streptococcus mutans* (*S. mutans*), studied their biological functions in microbial proliferation and biofilm formation, and further investigated the underlying mechanism. Fourteen hexapeptides were simulated, 13 of which were successfully synthesized. We found that the amyloid‐forming hexapeptides (AFhPs) displayed efficient broad‐spectrum antibiofilm activity against the Gram‐positive bacteria *S. mutans*, *Streptococcus sanguis* and *Staphylococcus aureus*, Gram‐negative bacteria *Escherichia coli* and fungus *Candida albicans*, by aggregating into rigid amyloid fibres agglutinating microbes, whereas the non‐amyloid‐forming hexapeptides (non‐AFhPs) did not. The AFhPs did not kill microbes and showed little or no cytotoxicity. Furthermore, a set of AFhPs displayed broad‐spectrum antibiofilm activity, regardless of its source. The microbial cell wall carbohydrates, peptidoglycan (PGN), lipoteichoic acid (LTA), glucan and zymosan A, mediated AFhP binding and triggered significant AFhP fibrillation. Although amyloid fibres agglutinated lipid membrane model – large unilamellar vesicles (LUVs) – and LUVs facilitated AFhP fibrillation, the roles of lipid membranes in AFhP antibiofilm activities remain to be elucidated. We highlight the potential use of AFhPs as novel antibiofilm agents.

## Graphical abstract

AFhPs aggregated into rigid amyloid fibers, which agglutinated microbes and exerted as antibiofilm agents. AFhPs have no microbicidal activity and little or no cytotoxicity. AFhPs may be utilized as novel antibiofilm agents.

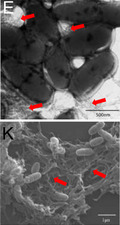

## Introduction

Amyloid fibres, referred to as fibrillar proteins or peptide aggregates with a cross‐β structure, were originally regarded as misfolded products that led to human disorders, including neurodegenerative diseases, diabetes and amyloidosis (Nizhnikov *et al*., 2015). However, during the last decade, increasing evidence has suggested that amyloid fibres exert multiple functional roles in processes including innate immunity, melanin synthesis, bacterial biofilm formation and bacterial virulence (Romero *et al*., 2010; Chu *et al*., 2012; Schwartz *et al*., 2012; Gallo *et al*., 2015; Nizhnikov *et al*., 2015; Bergman *et al*., 2016; Taglialegna *et al*., 2016a, 2016b,2016a, 2016b; Van Gerven *et al*., 2018). Moreover, amyloid‐forming proteins or peptides have been shown to have antimicrobial activities *in vivo*. Bednarska *et al*. (2016) hypothesized that amyloid‐forming peptides whose sequences were derived from amyloid regions of bacterial proteins could induce toxic protein aggregation more efficiently in bacteria. Furthermore, this group identified a set of short amyloid‐forming peptides simulated from the proteome of *Staphylococcus epidermidis* displaying antimicrobial activity. Human α‐defensin (HD) 6, secreted by Paneth cells of the small intestine, was also found to possess amyloid‐forming ability; besides, HD6 could only agglutinate microbes and lacked microbe‐killing activities (Chu *et al*., 2012). Short synthetic amyloid‐forming peptides with microbe agglutination but without microbe‐killing activities have not been hitherto reported.

*Streptococcus mutans* (*S. mutans*) is a prominent cause of dental caries (Ajdic *et al*., 2002). Recently, *S. mutans* was found to produce functional amyloid fibres emanating from its surface, playing important roles in biofilm formation (Oli *et al*., 2012). Cell‐surface‐localized adhesin Pac (aka AgI/II, P1) was the main amyloid‐forming protein of *S. mutans*. Truncated protein C123 (aka AgII) was reported to represent the amyloid‐forming moiety of Pac (Oli *et al*., 2012; Besingi *et al*., 2017). We hypothesized that simulating short amyloid‐forming peptides from a well‐known microbial amyloid protein would be more efficient and provide more accurate results. Hence, we simulated short amyloid‐forming peptides from C123 sequences and studied their effects on microbial proliferation and biofilm formation, and further investigated the possible underlying mechanisms.

## Results

### Simulations of hexapeptides using computational methods

**Fig. 1:**
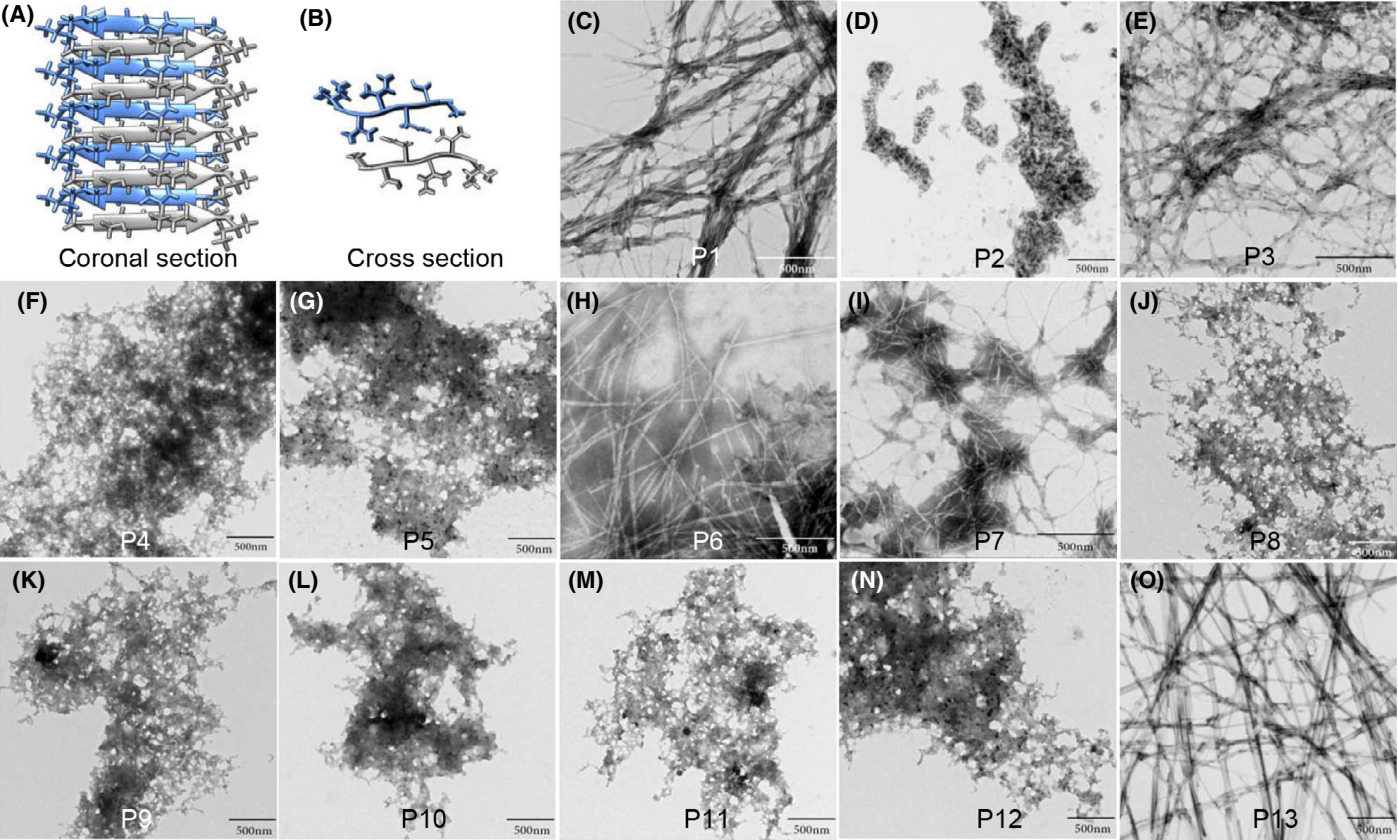
The simulated structure and aggregated morphology of 13 hexapeptides derived from the C123 sequence. A. The sagittal plane showed P3 canonical steric‐zipper architecture of β‐sheets composed of parallel β‐strands. B. The coronal plane showed the β‐strands were perpendicular to the fibril axis. C–O. Aggregation results of the 13 hexapeptides (P1 to P13) observed using TEM. Among them, P1, P3, P6, P7 and P13 aggregated into rigid amyloid fibres, while P2, P4, P5, P8, P9, P10, P11 and P12 aggregated into an amorphous morphology.

By using combined information from three computational methods, 14 hexapeptides with the ability to form amyloid fibres were simulated from C123 sequence, among which 13 hexapeptides were successfully synthesized (Table 1). The 13 hexapeptides were named P1 to P13 according to their simulated amyloid‐forming ability – Rosetta energy. All the 13 simulations were structural modelling, and only one hexapeptide P3 was shown here to show the simulated crystal structure. The simulated crystal structures of P3 showed a canonical steric‐zipper β‐sheet architecture composed of parallel β‐strands (Fig. 1A) and β‐strands perpendicular to the fibril axis (Fig. 1B).

**Table 1:** Short amyloid‐forming peptides derived from C123 sequence.

Name	Position	Sequence	Rosetta energy
P1	1319–1324	ASNIVI	−26.6
P2	1084–1089	ATLATF	−25.8
P3	1040–1045	TSFVLV	−25.3
P4	1104–1109	VVGQVL	−24.7
^a^	1083–1089	AATLAT	−24.6
P5	1480–1485	YSSNTV	−24.6
P6	1283–1288	GIDLKI	−23.7
P7	1399–1404	FAKVDI	−23.7
P8	1363–1368	LIGGII	−23.5
P9	1479–1484	TYSSNT	−23.5
P10	1277–1282	DTYVKT	−23.2
P11	1453–1488	AESYIQ	−23.1
P12	1400–1405	AKVDIT	−23
P13	1462–1467	IAVGTF	−23

^a^ Not successfully synthesized.

### The amyloid‐forming ability of the simulated hexapeptides

To ensure hexapeptides with amyloid‐forming ability aggregate thoroughly, we facilitated their aggregation in pH = 3 sterile double‐distilled water (ddH_2_O) at 60°C for 24 h, and the final concentrations of hexapeptides were 0.05 mg ml^−1^. Their aggregation states were observed by using transmission electron microscopy (TEM). Results (Fig. 1C–O) showed that only P1, P3, P6, P7 and P13 aggregated into rigid amyloid fibres. We named these hexapeptides amyloid‐forming hexapeptides (AFhPs). The other hexapeptides including P2, P4, P5, P8, P9, P10, P11 and P12, which aggregated into amorphous morphology, were named non‐AFhPs.

### AFhP monomers inhibit *S. mutans* biofilm formation without exhibiting microbicidal activity

**Fig. 2:**
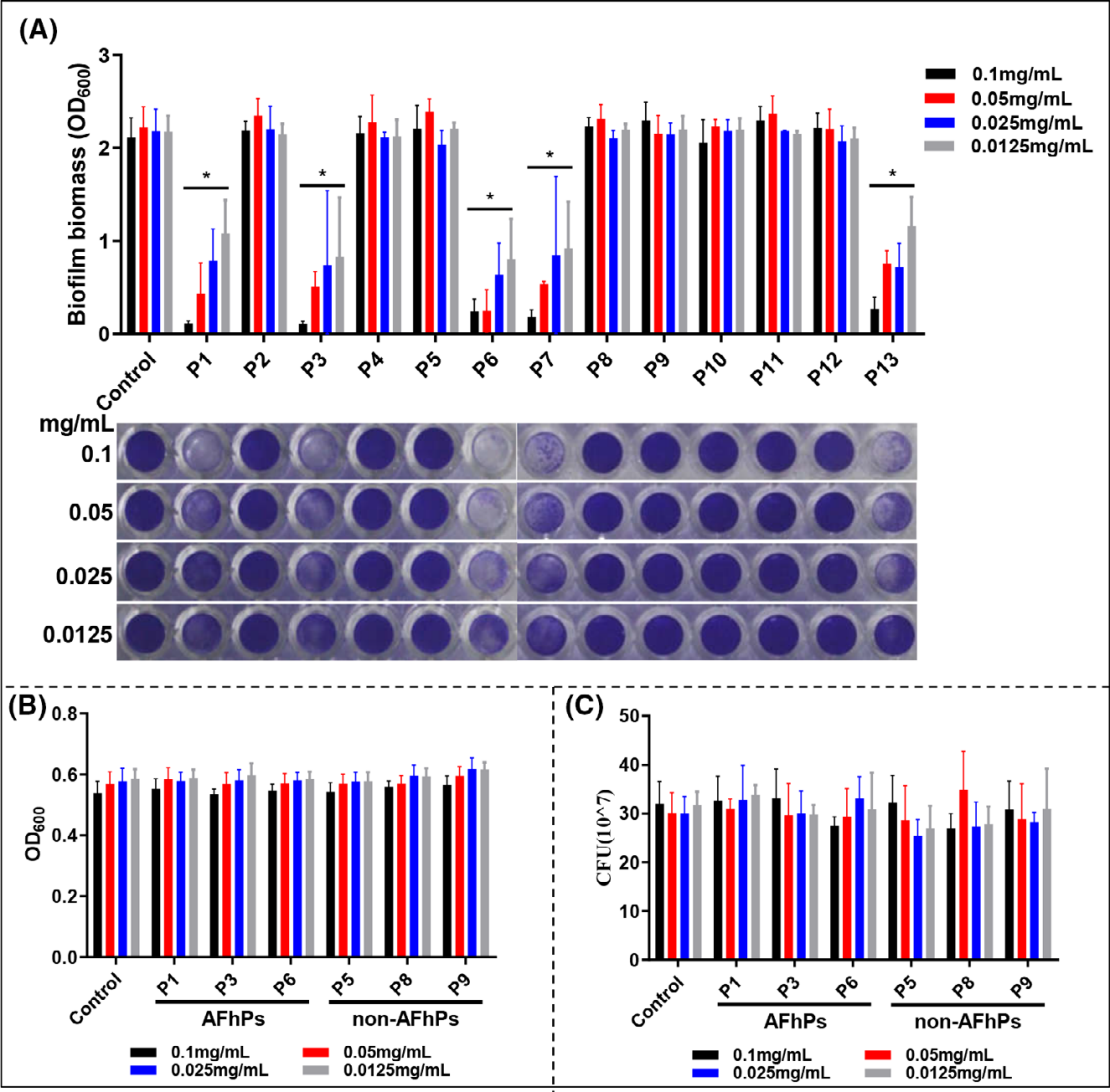
Effect of the 13 hexapeptides on *S. mutans* biofilm formation, planktonic *S. mutans* proliferation and living *S. mutans* cell number. A. The effect of the 13 hexapeptides on new *S. mutans* biofilm formation. P1, P3, P6, P7 and P13 showed a significant concentration‐dependent on biofilm formation inhibition, while P2, P4, P5, P8, P9, P10, P11 and P12 did not significantly affect new *S. mutans* biofilm formation. B. The AFhPs (P1, P3 and P6) and non‐AFhPs (P5, P8 and P9) at all the four tested concentrations (0.1/0.05/0.025/0.0125 mg ml^−1^) had no significant influence on planktonic *S. mutans* proliferation. C. Colony‐forming unit counting showed that AFhPs (P1, P3 and P6) and non‐AFhPs (P5, P8 and P9) at the four concentrations did not affect the number of living *S. mutans* cells. *P < 0.05; *n* = 3.

AFhPs (P1, P3, P6, P7 and P13) significantly impeded new *S. mutans* biofilm formation at all the tested concentrations (0.1, 0.05, 0.025 and 0.0125 mg ml^−1^). The mass of the *S. mutans* biofilm attached to the bottom of the 96‐well plate was dramatically decreased (*P* < 0.05), and the effect was concentration‐dependent, while the non‐AFhPs (P2, P4, P5, P8, P9, P10, P11 and P12) did not inhibit *S. mutans* biofilm formation (*P *> 0.05) (Fig. 2A).

Given the similar effects of hexapeptides on *S. mutans* biofilm formation, we randomly selected three AFhPs (P1, P3 and P6) and three non‐AFhPs (P5, P8 and P9) as representative hexapeptides for the following experiments. For confocal laser scanning microscopy (CLSM), TEM and scanning electron microscopy (SEM) experiments, all the three selected AFhPs and non‐AFhPs were investigated and documented, but only P3 and P8 images were presented to represent AFhP and non‐AFhP results respectively.

No significant differences in planktonic *S. mutans* proliferation values were observed among the control, AFhPs (P1, P3 and P6) and non‐AFhPs (P5, P8 and P9) groups at all the four tested concentrations (*P *> 0.05) (Fig. 2B). The plate counting assay neither showed any differences in the number of colony‐forming units between these groups at the four concentrations (*P *> 0.05) (Fig. 2C). Furthermore, effects of 0.05 mg ml^−1^ hexapeptides on the dead/live ratio of *S. mutans* were investigated by CLSM experiment. Three‐dimensional reconstruction CLSM images showed that the biovolume of *S. mutans* cells was obviously decreased in AFhP(P3) group when compared with control and non‐AFhP(P8) groups (Fig. S1A–C), and the calculated dead/live ratio of *S. mutans* cells showed no significant differences among control and AFhPs (P1, P3 and P6) groups and non‐AFhPs groups (P5, P8 and P9) (Fig. S1D). Since the effect of hexapeptide at 0.05 mg ml^−1^ was obvious and representative, a final concentration of 0.05 mg ml^−1^ was used in the following experiments.

### Cytotoxicity of AFhP monomers

Compared with the control group, AFhP(P1) and AFhP(P3) at 0.05 mg ml^−1^ slightly impeded normal oral mucosa keratinized epithelial cell (NOK) proliferation and the difference was significant (*P* < 0.05), whereas 0.05 mg ml^−1^ AFhP(P6) had no significant influence on NOK proliferation (*P *> 0.05). Furthermore, the non‐AFhPs (P5, P7 and P8) did not influence NOK proliferation (*P *> 0.05, Fig. S2).

### AFhP monomers inhibit *S. mutans* biofilm formation through fibrillation

**Fig. 3:**
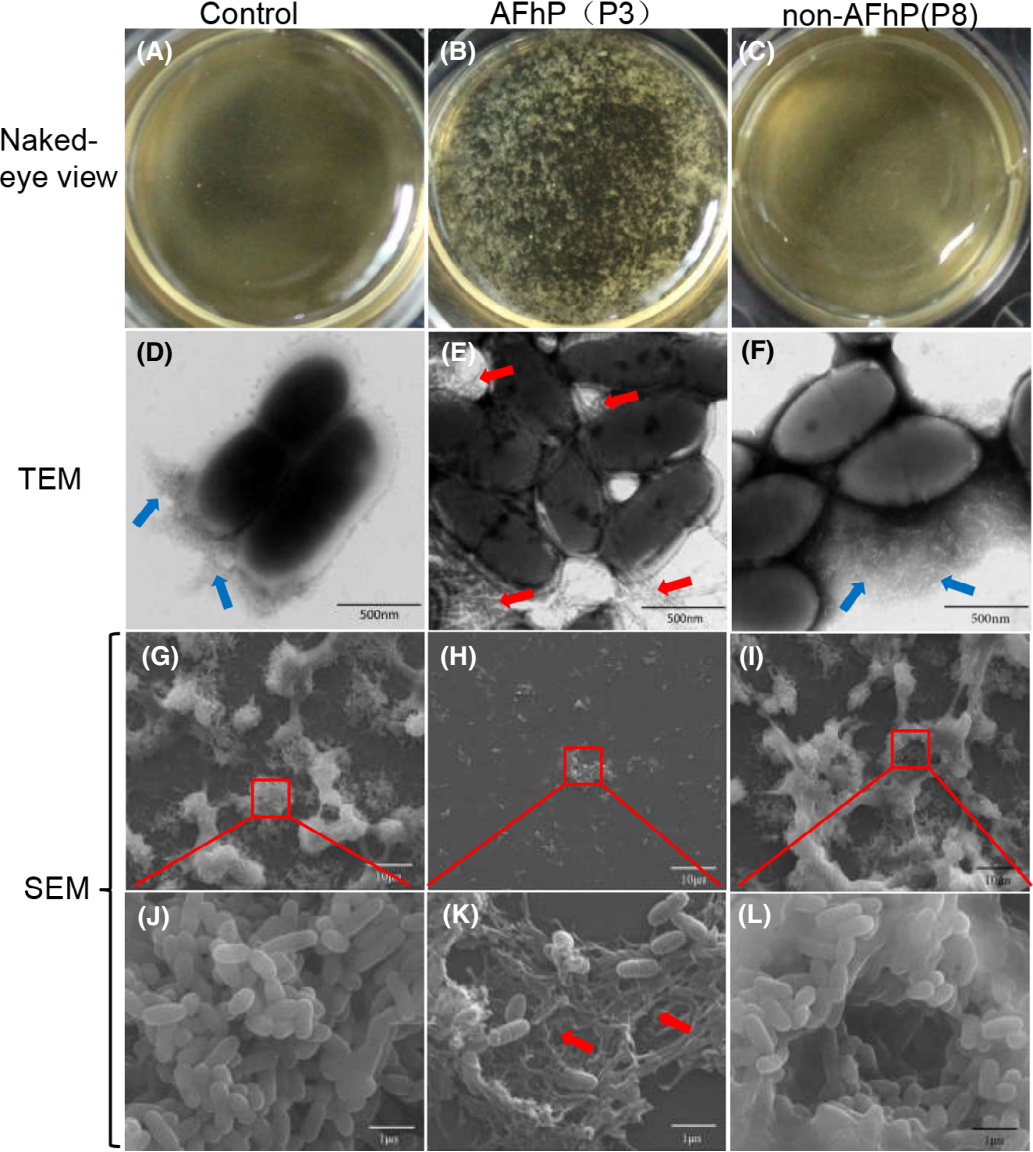
Observation of the effects of AFhPs and non‐AFhPs on *S. mutans* biofilm formation. A to C: The naked‐eye view of the 24 h‐cultured *S. mutans* biofilm treated by 0.05 mg ml^−1^ hexapeptides or not. The biofilm stuck to the bottom of the plate in the control (A) and non‐AFhP(P8)‐treated groups (C). Flocculent precipitate floating in the medium was seen in the AFhP(P3)‐treated group (B). D to F: TEM results of 24 h‐cultured *S. mutans* biofilm treated by 0.05 mg ml^−1^ hexapeptides or not. Twisted amyloid fibres produced by *S. mutans* could be seen in the control (D) and non‐AFhP(P8)‐treated group (F). A large amount of long rigid amyloid fibres agglutinating *S. mutans* cells were seen in the AFhP(P3)‐treated group. G to I: The 24 h‐cultured *S. mutans* biofilm treated by 0.05 mg ml^−1^ hexapeptides or not observed by SEM at low magnification. Mature *S. mutans* biofilms structure were seen in the control (G) and non‐AFhP(P8)‐treated group (I), while small *S. mutans* clusters were seen in AFhP(P3)‐treated group. J to L: Amplified images of G to I. *S. mutans* cells agglutinated by intertwined rigid amyloid fibres could be seen in the AFhP(P3)‐treated group (K), and structured *S. mutans* cells in biofilm could be seen in control (J) and non‐AFhP(P8)‐treated group (L). Red arrow: rigid amyloid fibres aggregated by AFhP(P3). Blue arrow: twisted amyloid fibres produced by *S. mutans*.

Flocculent precipitates floating in the medium could be seen grossly in the 0.05 mg ml^−1^ AFhP(P3)‐treated 24 h‐cultured *S. mutans* biofilm group, while biofilm in the control and non‐AFhP(P8) group stuck tightly to the bottom of the plates (Fig. 3A–C). We collected the *S. mutans* cells from biofilm, stained and viewed them using TEM. The short twisted amyloid fibres produced by *S. mutans* itself could be seen in the control (Fig. 3D) and non‐AFhP(P8) group (Fig. 3F), while large amounts of long and rigid amyloid fibres, instead of short twisted amyloid fibres, agglutinating *S. mutans* cells were seen in the AFhP(P3) group (Fig. 3E). Moreover, long, thick and rigid amyloid fibres agglutinating *S. mutans* cells surface were sometimes seen (Fig. S3). TEM results of the other AFhPs (P1 and P6) and non‐AFhPs (P5 and P9) were similar to that of AFhP(P3) and non‐AFhP(P8) respectively (Fig. S4). These data indicated that the added AFhP monomers aggregated into rigid amyloid fibres, which agglutinated *S. mutans* cells into clusters and thus inhibited new biofilm formation. As the twisted amyloid fibres produced by *S. mutans* could not be seen using SEM, we used SEM to observe the 24 h‐cultured *S. mutans* biofilms. *S. mutans* biofilms in control (Fig. 3G) and non‐AFhP(P8)‐treated (Fig. 3I) group were dense and continuous when observed at low magnification, while small *S. mutans* biofilm clusters were sparsely scattered in the 0.05 mg ml^−1^ AFhP(P3)‐treated group (Fig. 3H). When viewed at higher magnification, *S. mutans* cells formed a mature three‐dimensional structure biofilm in control (Fig. 3J) and non‐AFhP(P8) group (Fig. 3L), while *S. mutans* cells were seen entwined and linked by rigid amyloid fibres in AFhP(P3) group (Fig. 3K).

It was noticeable that there were also more flocculent precipitates in the 0.05 mg ml^−1^ AFhP(P3)‐treated planktonic *S. mutans* group than in control or non‐AFhP(P8)‐treated group (Fig. S5A). When viewed using TEM, rigid amyloid fibres were also found agglutinating *S. mutans* surface in the AFhP(P3)‐treated group (Fig. S5B). To determine the amount of time that AFhPs agglutinated *S. mutans* cells, we added 0.05 mg ml^−1^ AFhP(P3) to an inactivated *S. mutans* solution (1 × 10^9^ CFU ml^−1^), and it was encouraging that AFhP(P3) agglutinated *S. mutans* cells immediately and made the solution clear within 10 min.

### Aggregated AFhP fibres maintain their antibiofilm activity

**Fig. 4:**
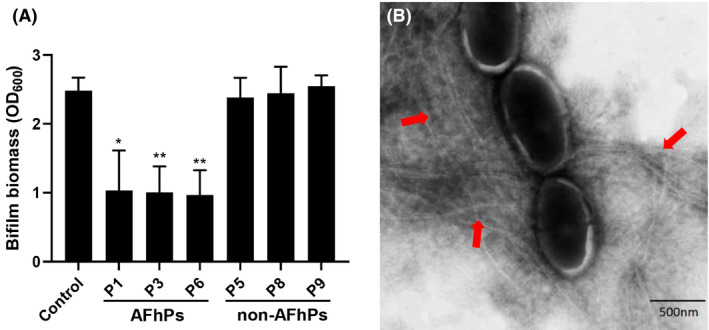
Effect of aggregated AFhPs on *S.mutans* biofilm formation. A. Crystal violet assay showed that 0.05 mg ml^−1^ AFhP (P1, P3 and P6) fibres could significantly decreased the biofilm mass (*P *< 0.05), while 0.05 mg ml non‐AFhP (P5, P8 and P9) fibres did not affect *S. mutans* biofilm formation. B. TEM results of the pre‐aggregated AFhP(P3) fibres on *S. mutans* biofilm formation. The pre‐aggregated AFhP(P3) fibres could also agglutinate *S. mutans*. Red arrow: pre‐aggregated rigid amyloid fibres by AFhP(P3); *P < 0.05, **P < 0.01; *n* = 3.

We first facilitated AFhP monomers into amyloid fibres. Although the pre‐aggregated amyloid fibres were insoluble in ddH_2_O, no flocculent precipitates could be seen grossly in solution, and the successfully aggregated AFhP fibres were verified using TEM and thioflavin T (ThT) assays. After that, the pre‐aggregated AFhP fibres were added into the *S. mutans* biofilm culture medium and the finial concentration of AFhPs were 0.05 mg ml^−1^. Results showed that AFhP fibres also inhibited new *S. mutans* biofilm formation and possessed antibiofilm activity (*P* < 0.05, Fig. 4A). TEM images showed that *S. mutans* cells were agglutinated by rigid amyloid fibres (Fig. 4B).

### AFhPs exhibited broad‐spectrum antibiofilm action

We examined two additional hypotheses. The first was whether AFhPs derived from C123 sequence had broad‐spectrum antibiofilm action, and the second was whether AFhPs simulated from other species genomes could also exhibit antibiofilm activity.

**Fig. 5:**
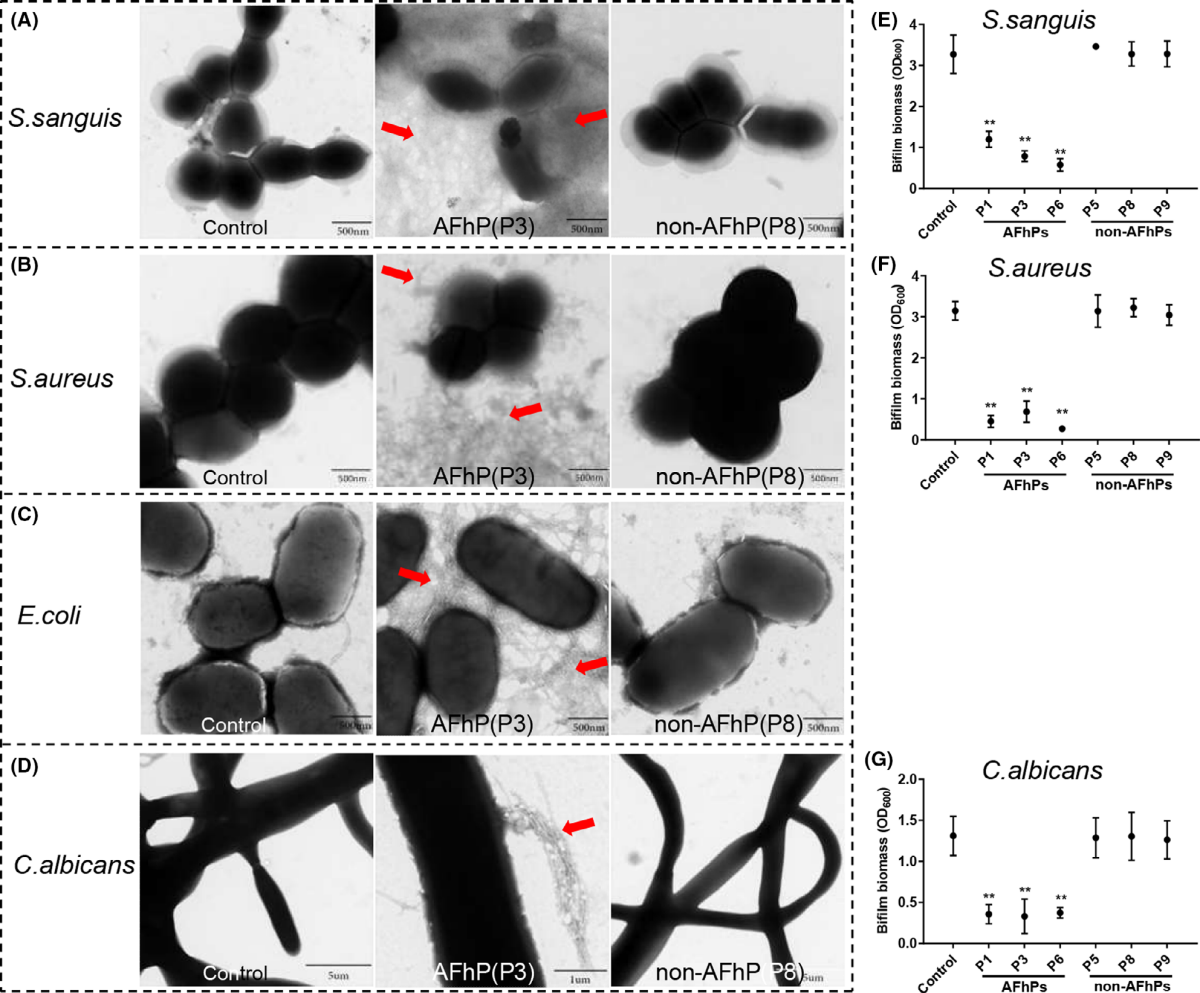
Effect of AFhPs and non‐AFhPs derived from the C123 sequence on biofilm formation of other microbes. A–D. Compared with the control and non‐AFhP(P8)‐treated group, 0.05 mg ml^−1^ AFhP(P3) formed rigid amyloid fibres that agglutinated the Gram‐positive bacteria *Streptococcus sanguis* (*S. sanguis*) and *Staphylococcus aureus* (*S. aureus*), Gram‐negative *Escherichia coli* (*E. coli*) bacteria and the fungus *Candida albicans* (*C. albicans*). E–G. Compared with the control and non‐AFhPs‐treated groups, 0.05 mg ml^−1^ AFhPs significantly decreased the biofilm biomass of *S. sanguis*, *S. aureus* and *C. albicans*. Red arrow: rigid amyloid fibres aggregated by AFhP(P3); **P < 0.01; *n* = 3.

We selected the Gram‐positive bacteria *S. sanguis* and *S. aureus,* the Gram‐negative bacteria *E. coli*, and the fungus *C. albicans* to examine whether AFhPs from C123 sequence had broad‐spectrum antibiofilm action. Results showed that 0.05 mg ml^−1^ AFhP(P3) monomers aggregated into rigid amyloid fibres agglutinating Gram‐positive bacteria, Gram‐negative bacteria and fungus, while the morphologies of microbes were normal when compared with control and non‐AFhP(P8) group (Fig. 5A–D). The new formed biofilm biomass was dramatically decreased in the AFhPs‐treated groups for all the tested species when compared with control group, while non‐AFhPs did not affect biofilm biomass (Fig. 5E–G). Biofilm formed by *E. coli* was too fragile and easily washed down, and thus did not satisfy the criteria for the crystal violet assay, so the statistical analyses of the *E. coli* biofilm biomass results were not performed.

A concentration of 0.05 mg ml^−1^ AFhPs also deposited planktonic microbes (*S. sanguis, S. aureus, E. coli* and *C. albicans*) into larger flocculent precipitates comparing with control and non‐AFhPs groups (Fig. S6A–D). TEM results revealed that AFhPs aggregated into rigid amyloid fibres agglutinating planktonic Gram‐positive and Gram‐negative bacteria, and fungus cells (Fig. S6E–H). Moreover, AFhPs at 0.05 mg ml^−1^ did not affect the proliferation values of the tested microbes (Fig. S6I–L).

In ZipperDB, there was a database that contained predictions for some proteins in different species genomes. To examine our second hypothesis, species of homo sapiens, rattus rattus and HIV were selected, and we selected the sequence with the highest amyloid‐forming ability (with the lowest Rosetta energy) from the first predicted proteins in ZipperDB. The sequences were GQSIAI, SSHMCM, and NQSVSI respectively. TEM verified that only GQSIAI could aggregate into rigid amyloid fibres (Fig. S7A–C). GQSIAI could also significantly impede *S. mutans* biofilm formation (*P* < 0.01, Fig. S7D) by forming amyloid fibres agglutinating *S. mutans* (Fig. S7E) without affecting planktonic *S. mutans* proliferation (*P *> 0.05, Fig. S7F), in accordance with our previous findings.

### Microbial cell wall carbohydrates mediate AFhP binding and fibrillation

**Fig. 6:**
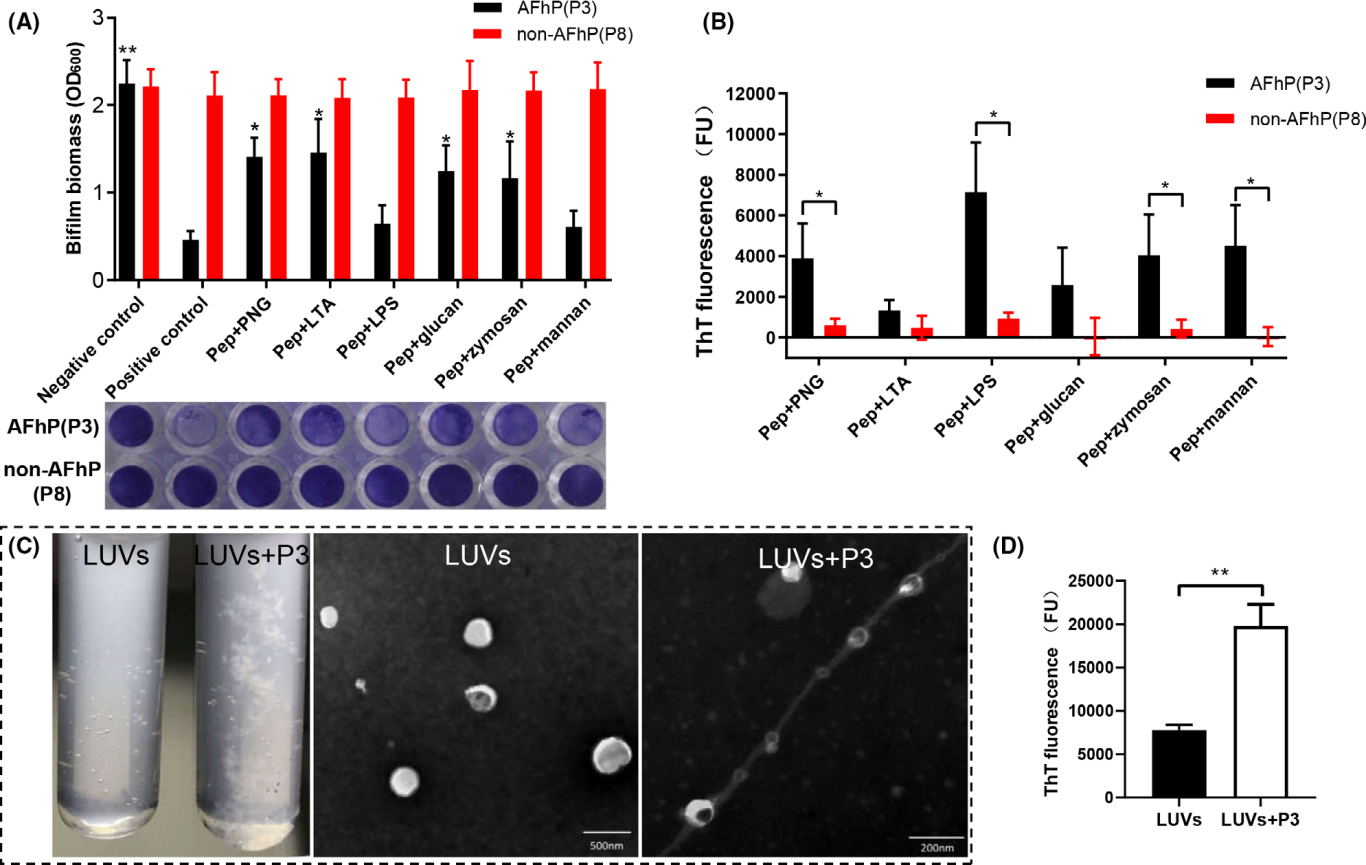
Mechanism exploration of cell wall carbohydrates and LUVs on AFhP binding and fibrillation. A. Compared with the positive control group, 0.05 mg ml^−1^ AFhP(P3)‐treated group, P3 + PNG/LTA/glucan/zymosan significantly enhanced the *Streptococcus mutans* (*S. mutans*) biofilm biomass, while P3 + LPS/mannan had no influence on it. B. The ThT fluorescence assay results showed that the addition of PGN, LPS, zymosan A and mannan significantly enhanced AFhP(P3) fluorescence intensity, compared with the non‐AFhP(P8) group. C. When AFhP(P3) was added to the LUV solution, a flocculent precipitate formed. TEM results showed that AFhP(P3) aggregated into amyloid fibres and agglutinated LUVs. D. The ThT fluorescence assay showed that the addition of LUVs significantly enhanced fluorescence intensity, comparing with LUVs group. *P < 0.05, **P < 0.01; *n* = 3.

We investigated whether AFhP fibres agglutinated microbes via microbial cell wall carbohydrates to explore the underlying mechanism. Compared with the positive control 0.05 mg ml^−1^ AFhP(P3) group, AFhP(P3)+PNG/LTA/glucan/zymosan significantly enhanced *S. mutans* biofilm biomass (*P* < 0.05, Fig. 6A), while AFhP(P3)+LPS/mannan had no influence on biofilm biomass (*P > *0.05), indicating that PGN, LTA, glucan and zymosan significantly attenuated the AFhP(P3) antibiofilm action. We further characterized the influence of microbial cell wall carbohydrates on AFhP fibrillation using the ThT assay. ThT fluorescence assay results showed that the addition of PGN, LPS, zymosan A and mannan significantly enhanced AFhP(P3) fluorescence intensity, compared with the corresponding non‐AFhP(P8) groups (*P *< 0.05, Fig. 6B).

### Large unilamellar vesicle (LUV) model membranes facilitate AFhP fibrillation

Lipid bilayer membranes were reported to be related to amyloid protein fibrillation; hence, we further studied the effect of lipid bilayer membranes on AFhP fibrillation. We prepared a 1‐palmitoyl‐2‐oleoyl‐sn‐glycero‐3‐phosphocholine (POPC) and 1‐palmitoyl‐2‐oleoyl‐sn‐glycero‐3‐phospho‐(1‐rac‐glycerol) (POPG) mixture of LUV model membranes to imitate the microbial lipid bilayer membrane. After adding AFhP(P3) to LUVs, flocculent precipitates formed within 2 h (Fig. 6C). We further observed the morphology of AFhP(P3) fibres prepared in the presence of LUVs, and results showed that amyloid fibres agglutinated LUVs (Fig. 6D). The ThT fluorescence intensity was greatly enhanced upon addition of AFhP(P3) to LUVs (*P* < 0.01, Fig. 6E), indicating that LUVs could facilitate AFhP fibrillation.

## Discussion

Recently, amyloid fibres have been found to play important roles in the microbial field. Amyloid fibres produced by microbes mediated microbial adhesion, biofilm formation and simulating autoimmunity; however, amyloid fibres aggregated by peptides or proteins were reported to exert antimicrobial activities (Bergman *et al*., 2016). In our study, we found that AFhPs displayed efficient broad‐spectrum antibiofilm action by aggregating into rigid amyloid fibres agglutinating pathogens. Thus, these AFhPs may be utilized as novel antibiofilm agents. AFhPs impeded all biofilm formation of Gram‐positive and Gram‐negative bacteria, and fungus, and rapidly agglutinated planktonic microbes into flocculent precipitates. Bacterial agglutination would prevent the spread of infection and facilitate bacterial clearance by the immune cells of the host, which was appealing (Gorr *et al*., 2008). Another notable finding in our study was that AFhPs displayed broad‐spectrum antibiofilm activity regardless of its resource, suggesting that there was a set of unsimulated AFhPs displaying broad‐spectrum antibiofilm activities.

The proliferation OD values, plate counting assay and CLSM experiments showed that AFhPs had no effect on the viability of microbes, indicating that the mechanism underlying AFhP antibiofilm activity did not involve microbe killing. A critical clue for understanding the mechanism of AFhP antibiofilm activity was obtained from the TEM and SEM results. TEM and SEM images showed that rigid amyloid fibres formed, which agglutinated microbes, leaving the morphologies of the microbes intact, indicating that amyloid fibres bound to the microbial surface. A similar phenomenon was observed for some amyloid peptides; nevertheless, the reported mechanisms were quite different. Chu *et al*. (2012) reported that HD6 was unlikely to target microbial surface carbohydrates because it lacked lectin‐like activity, and they found the predominant proteinaceous surface appendages of *Salmonella Typhimurium* flagella and type I fimbriae were the anchorage of HD6, and flagella and fimbriae also triggered HD6 amyloid nanonet formation. However, the microbes tested in our study, for example *S. sanguis* and *C. albicans,* lacked proteinaceous surface appendages. Therefore, proteinaceous surface appendages were unlikely to be the binding targets of AFhPs. Kumar *et al*. (2016) found that Aβ contained the heparin‐binding motif (XBBXBX) between residues 12 and 17, and suggested that Aβ adhesion inhibition and agglutination activities were mediated by lectin‐like carbohydrate binding. They found that mannan and glucan significantly attenuated Aβ’ adhesion inhibition and agglutination activities. Although the sequences of AFhPs in our study did not satisfy the regularity of XBBXBX, cell wall carbohydrates (PGN, LTA, glucan, and zymosan A) still significantly impeded AFhPs’ antibiofilm activities, suggesting that microbial cell wall carbohydrates are the binding targets. In the amyloid hypothesis of Alzheimer’s disease, the cytotoxicity of Aβ was supposed to be the result of interactions between Aβ fibrillation and cell lipid membranes (Niu *et al*., 2018; Fanni *et al*., 2019). Studies have verified that lipid membranes play a pivotal role in triggering and accelerating Aβ fibrillation (Lindberg *et al*., 2017). In this study, we found that LUVs noticeably accelerated AFhP fibrillation and clinging of LUVs to AFhP fibres. Nevertheless, it did not suggest that AFhP fibres bind to microbial lipid membranes. Our data indicated that rigid amyloid fibres were more likely to agglutinate microbes through touching microbial cell wall. The following evidence also provided clues that cell wall carbohydrates instead of lipid membranes were the binding targets of AFhPs. 1) Carbohydrates were the outermost structure of the microbial cell wall. 2) Carbohydrates of PGN layers in the Gram‐positive bacterial cell wall could permeate particles of approximately 2 nm or globular hydrophilic molecules of a maximum of 50 kDa that did not bind to PGN (Malanovic and Lohner, 2016). Amyloid fibres were large protein aggregates with a diameter of ~ 12 nm and a high molecular weight greater than 50 kD (Schwartz *et al*., 2012; Taglialegna *et al*., 2016a, 2016b,2016a, 2016b). For Gram‐positive bacteria, it was impossible for amyloid fibres to penetrate thick PGN layers to bind lipid membranes. Hence, the role of the lipid membrane in the microbial agglutination activities of AFhPs requires further investigation.

Amyloid‐forming peptides with both microbe agglutination and antimicrobial activities, such as amyloidogenic Aβ variant peptides and eosinophil cationic protein (ECP), have been previously reported. Amyloidogenic Aβ variant peptides, but not the non‐amyloidogenic peptides, can bind to microbial surfaces and cause large microbial agglutinates. In addition, amyloidogenic Aβ variant peptides were found to exert a direct antimicrobial activity against Gram‐positive and Gram‐negative bacteria, and fungus, but the mechanisms underlying these effects were not investigated (Spitzer *et al*., 2016). ECP was reported to bind to the Gram‐negative bacterial surface favoured by interactions with the LPS in the outer membrane (Torrent *et al*., 2008; Pulido *et al*., 2012). Moreover, previous studies have revealed a link between amyloid peptides and antimicrobial activities (Kagan *et al*., 2012; Zhang *et al*., 2014; Zheng *et al*., 2020). Different mechanisms underlying the amyloid‐forming peptides’ antimicrobial activities were also proposed. Most of them involved the formation of amyloid ion channels in lipid membranes, similar to the ion channels formed by antimicrobial peptides (AMPs), which caused toxic leakage of cellular constituents (Kagan *et al*., 2012). Bednarska *et al*. (2016) found that amyloid‐forming peptides accumulated inside microbes and induced intracellular aggregation, disturbing the process of cell division. However, the AFhPs simulated in our study only had microbial agglutination ability but no antimicrobial activities. The explanation for the different functions of the amyloid‐forming peptides is unclear. Previous study showed that amyloid fibres formed *in vitro* by 40‐ and 42‐residue amyloid‐β peptides (Aβ40 and Aβ42) were polymorphic, and variations in structure *in vivo* correlated with different Alzheimer's disease phenotype (Qiang *et al*., 2017). We also noticed that the amyloid fibres produced by *S. mutans* were ‘soft’ and related to *S. mutans* adhesion and biofilm formation, while amyloid fibres aggregated by AFhPs were rigid and they showed antibiofilm activities (Chen *et al*., 2019). Thus, we hypothesized that the different functions of amyloid‐forming peptides might result from different amyloid structures that they formed. Besides, the different functions might also be related to a substantial sequence‐specific characteristic of amyloid‐forming peptide. A growing evidence suggested that many different peptides could aggregate into amyloid fibres, and amino acid side‐chains dramatically influenced fibre formation, leading to a suggestion that amyloid fibres were primitive main chain‐dominated structure (Klein *et al*., 2018). However, no sequence conservation has been found among different amyloid‐forming proteins or proteins up to now. Future studies need to focus on amyloid structure and peptide sequence characteristic to find out the underlying mechanism.

Most amyloidogenic peptides are highly hydrophobic, introducing challenges to synthesis (Chemuru *et al*., 2014). In this study, good yield and purity could be obtained for most hexapeptides in the solid‐phase synthesis at different times, but few hexapeptides are unstable at different time synthesis, e.g. P1 and P10. There once synthesis, P1 neither aggregated into amyloid fibres nor exerted antibiofilm activity, which also indirectly proved that hexapeptide with amyloid‐forming ability exerted antibiofilm action.

Overall, our work presents evidence that AFhPs inhibit biofilm formation in Gram‐positive and Gram‐negative bacteria, and fungus by aggregating into rigid amyloid fibres that entangle microbe cells. To the best of our knowledge, AFhPs are the shortest synthetic peptides with highly efficient antibiofilm activities but without antimicrobial activities; hence, we highlight the potential use of AFhPs as novel antibiofilm agents.

## Experimental procedures

### Computational simulation of short amyloid‐forming peptides

Short amyloid‐forming peptides were simulated from the C123 sequence using three computational methods, namely ZipperDB, Tango and Waltz (Fernandez‐Escamilla *et al*., 2004; Goldschmidt *et al*., 2010; Maurer‐Stroh *et al*., 2010). Among the three computational methods, ZipperDB could analyse the amyloid‐forming ability of every six sequence and provided a Rosetta energy value. When the value of Rosetta energy < −23 kcal mol^−1^, it indicated the sequence could form amyloid fibres theoretically. Short peptides from C123 sequence were selected when at least two computational methods showed amyloid‐forming ability. ZipperDB also provided predictions for some proteins in different species genomes. Species of Homo sapiens, rattus rattus and HIV were selected, and we chose the first predicted proteins and select the sequence with the highest amyloid‐forming ability (with the lowest Rosetta energy).

### Peptide synthesis and storage

All hexapeptides used in this work were synthesized at > 95% purity by Genscript (Nanjing, China) following the solid‐phase peptide synthesis protocol. All hexapeptides were modified by N‐terminal acetylation and C‐terminal amidation. Lyophilized peptide powder was stored or diluted in 10% dimethylsulphoxide (DMSO) to a stock concentration of 1 mg ml^−1^ at −20°C.

### Microbial stains and growth conditions

*Streptococcus mutans* UA159 (ATCC 700610™), *Streptococcus sanguis* (ATCC 10556), *Staphylococcus aureus* (ATCC 6538p), *Escherichia coli* (ATCC 8739) and *Candida albicans* (SC5314) were used in this study. *S. mutans* and *S. sanguis* were incubated statically in brain heart infusion broth media (BHI) at 37°C for 24 h under anaerobic conditions (85% N_2_, 10% H_2_, 5% CO_2_) to obtain planktonic bacteria growth to plateau stage, and in BHI + 1% sucrose for 24 h to obtain a mature biofilm. Planktonic *S. aureus* and *E. coli* were cultivated in tryptic soy broth (TSB) at 37°C for 24 h, and *C. albicans* was cultivated in Sabouraud medium with shaking at 200 rpm at 37°C for 24 h. For biofilm formation, *E. coli* was cultured in 0.5 g l^−1^ yeast extract and 10 g l^−1^ casamino acids for 24 h, *S. aureus* in TSB with 3% NaCl and 0.5% glucose for 24 h, and *C. albicans* in artificial saliva with 10% fetal bovine serum (FBS) and 1% sucrose for 24 h.

### Planktonic microbe proliferation, plate counting and crystal violet assays

Hexapeptides at different concentrations (0.1, 0.05, 0.025 and 0.0125 mg ml^−1^) were first added to their corresponding medium before culturing biofilm or planktonic microbes. The starting ODs of the solution containing microbes, medium and hexapeptides were relatively stable, which were about 0.10 for *S. mutans* and *S. sanguis*, 0.15 for *S. aureus* and *E. coli,* and 0.40 for *C. albicans* respectively.

Crystal violet assay was performed as described previously (Chen *et al*., 2019). Briefly, microbes were added into their corresponding biofilm culture medium with hexapeptides and incubated in 96‐well plates for 24 h. The control group were added with the same concentration of DMSO as the hexapeptide groups. The new formed biofilm was washed twice with PBS, fixed with absolute methanol for 15 min, air‐dried for at least 15 min, stained with 0.1% crystal violet for 15 min and washed with ddH_2_O until no excess dye present, and then, the absorbance was measured using a spectrophotometer at OD_600_. The mass of *E. coli* biofilm was not determined because the mature *E. coli* biofilm was not stable and very easily roused out.

Planktonic microbes were incubated in 2 ml Eppendorf tubes for 24 h with their corresponding medium containing hexapeptides or not. After that, the planktonic microbe solution was mixed well before being added to 96‐well plates. The amount of planktonic microbe proliferation was detected using a spectrophotometer at OD_600_, and the residual planktonic *S. mutans* solution treated by 0.05 mg ml^−1^ was diluted for plate counting assay. After culturing for 24 h in BHI agar plates, the colony‐forming units (CFUs) were calculated.

### Confocal laser scanning microscopy

*Streptococcus mutans* cultured in 1% BHIs with or without 0.05 mg ml^−1^ hexapeptides were incubated on confocal microscopy dishes for 24 h for biofilm formation. After that, biofilm was washed twice with sterile PBS and then dyed with the live/dead stains SYTO9 and propidium iodide (PI) for 15 min. The excitation wavelengths for SYTO9 and PI were 488 nm and 543 nm respectively. The ratio of dead/live bacteria was calculated using the comstat2.1 image‐processing software.

### Transmission electron microscopy

The hexapeptides (0.05 mg ml^−1^) were cultured at pH = 3 at 60°C for 24 h to ensure that the peptides with amyloid‐forming ability aggregated thoroughly. The 10 μl aggregated peptide solution was deposited on formvar/carbon‐coated nickel grids for 2 min, and negative staining was performed using 3% phosphotungstic acid for 2 min. Observations were made using a Hitachi TEM system.

### Scanning electron microscopy

*Streptococcus mutans* were grown on sterile microscopy slides in 24‐well plates. *S. mutans* were cultured in 1% BHIs with or without 0.05 mg ml^−1^ hexapeptides for 24 h for biofilm formation. The *S. mutans* biofilm on the slides was washed twice with ddH_2_O, fixed with 2.5% glutaraldehyde for 3 h and dehydrated using an alcohol gradient (30%, 50%, 70%, 90% and 100%). Subsequently, biofilm was freeze‐dried and then sputter‐coated with gold. Observations were made using a thermal field emission environmental SEM/EDS/EBSD system.

### Cell viability

The cytotoxicity of 0.05 mg ml^−1^ hexapeptides was evaluated in human NOKs using the cell counting kit‐8 (CCK‐8). NOKs were seeded in 96‐well plates at a density of 1 × 10^4^ cells/well and cultured in keratinocyte serum‐free medium (Invitrogen, Grand island, NY, USA) containing 0.05 mg ml^−1^ hexapeptides for 24 h at 37°C. The medium was replaced with keratinocyte serum‐free medium (100 µl) containing CCK‐8 (10 μl), and the cells were cultured for 2 h at 37°C. Absorbance was measured at 495 nm, and cell viability was assessed based on the absorbance of each sample relative to that of the control.

### Assay for determining the effect of microbial cell surface carbohydrates on the antibiofilm activity of the hexapeptides

PGN and LTA from Gram‐positive *S. aureus* bacteria, LPS from Gram‐negative *E. coli* bacteria as well as glucan, zymosan A, and mannan from *Saccharomyces cerevisiae* were purchased from Sigma. All the above microbial cell surface carbohydrates were diluted in ddH_2_O to a concentration of 1 mg ml^−1^. The final concentration of 0.05 mg ml^−1^ hexapeptides was preincubated for 1 h with microbial cell surface carbohydrates at a mass ratio of 1:1 before being added to *S. mutans* biofilm culture medium. After the cells were cultured for 24 h, the mass of the *S. mutans* biofilm formed was detected using the crystal violet assay.

### ThT fluorescence assay

The hexapeptides were mixed with microbial cell surface carbohydrates at a mass ratio of 1:1, and the final concentration of hexapeptides was 0.05 mg ml^−1^. ThT was added to the mixture at a final concentration of 20 μM. The mixture’s fluorescence was detected within 30 min by using a Multiskan Spectrum Spectrophotometer (Synergy H1) at an excitation of 430 nm and emission of 490 nm. The ThT fluorescence of hexapeptide fibrillation was calculated using the formula F_peptide+carbohydrates_–F_peptide_–F_Pcarbohydrates_ + F_buffer_.

### Assay for determining the effect of LUVs on hexapeptide adhesion and fibrillation

POPC and POPG were purchased from Avanti Polar Lipids (Alabaster, AL). Briefly, POPC and POPG were premixed at a molar ratio of 7:3, and POPC and POPG lipid mixtures (10 µM) were dissolved in 1 ml chloroform and methanol (v/v, 3:1). POPC and POPG films were obtained by drying the solution in a stream of dry nitrogen, followed by desiccation for at least 2 h. Sodium phosphate buffer (20 mM, pH 7.5) was added to the POPC and POPG films, which were subsequently vortexed to become rehydrated. An ultrasonic cell disruptor was used to sonicate the liquid for 30 min (working 5 s, interval 3 s, amplitude 40) to obtain LUVs. The hexapeptides were added to the LUV solution, the mixed solution was shaken at 200 rpm for 2 h, and the final concentration of hexapeptides was 0.05 mg ml^−1^. LUV solution without hexapeptides was set as the control group. TEM was used to detect the morphology, and the fluorescence assay, to detect the aggregation state of the hexapeptides.

## Conflict of interest

No conflicts of interest to declare.

## Supporting information


**Fig. S1.** Three‐dimensional reconstruction CLSM images of *S. mutans* biovolume and the calculated dead/live ratio of *S. mutans* cells. A to C: CLSM images showed that the biovolume of *S. mutans* cells were obviously decreased in AFhP(P3) group (B) when compared with control (A) and non‐AFhP(P8) group (C). D: No significant differences were found in the dead/live ratio of *S. mutans* cells among control, AFhPs (P1, P3, and P6) groups and non‐AFhPs groups (P5, P8 and P9). *n* = 3.
**Fig. S2.** Cell cytotoxicity of AFhPs and non‐AFhPs. CCK‐8 assay showed that AFhP(P1) and AFhP(P3) at 0.05 mg ml^−1^ significantly decreased NOKs proliferation, while AFhP(P6) and non‐AFhPs (P5, P8 and P9) at 0.05 mg ml^−1^ had no significant influence on NOKs proliferation. *P < 0.05; *n* = 3.
**Fig. S3.** AFhP(P3) at 0.05 mg ml^−1^ aggregated into long and thick rigid amyloid fibers, agglutinating *S. mutans* cells surface.
**Fig. S4.** TEM images of AFhPs and non‐AFhPs on *S. mutans* biofilm formation. A and B: AFhP(P1) and AFhP(P6) at 0.05 mg ml^−1^ formed rigid amyloid fibers agglutinating *S. mutans* cells, respectively. C and D: Twisted amyloid fibers produced by *S. mutans* itself could be seen in non‐AFhP(P5) and non‐AFhP(P9) groups. Red arrow: rigid amyloid fibers aggregated by AFhPs; Blue arrow: twisted amyloid fibers produced by *S. mutans*.
**Fig. S5.** Effect of AFhP and non‐AFhP on planktonic *S.mutans*. A: Naked eye view of the 24 h‐cultured planktonic *S. mutans*. There were more flocculent precipitates in 0.05 mg ml^−1^ AFhP(P3) treated group when comparing with control and 0.05 mg ml^−1^ non‐AFhP(P8) treated group. B: TEM results showed that AFhP(P3) aggregated into rigid amyloid fibers agglutinating *S. mutans*. Red arrow: rigid amyloid fibers aggregated by AFhP(P3).
**Fig. S6.** Effect of AFhPs and non‐AFhPs on other planktonic microbes proliferation. A to D: Naked eye view of the 24 h‐cultured planktonic G^+^ bacteria (*S. sanguis* and *S. aureus*), G^−^ bacteria (*E. coli*) and fungus (*C. albicans*) treated by 0.05 mg ml^−1^ hexapeptides or not. More flocculent precipitates were observed in AFhP(P3)‐treated group. E to H: TEM results showed that 0.05 mg ml^−1^ AFhP(P3) aggregated into rigid amyloid fibers agglutinating *S. sanguis*, *S. aureus*, *E. coli* and *C. albicans*. I to L: AFhPs and non‐AFhPs at a concentration of 0.05 mg ml^−1^ had no influence on the proliferation values of *S. sanguis,* *S. aureus*, *E. coli* and *C. albicans*. Red arrow: rigid amyloid fibers aggregated by AFhPs.
**Fig. S7.** Effect of hexapeptides derived from other species on *S.mutans* biofilm formation. A to C: The aggregated status of 0.05 mg ml^−1^ GQSIAI (A), SSHMCM (B) and NQSVSI (C) observed by TEM. GQSIAI could aggregate into rigid amyloid fibers, while SSHMCM and NQSVSI could not. D: GQSIAI at 0.05 mg/mL significantly decreased *S. mutans* biofilm biomass, while SSHMCM and NQSVSI did not. E: GQSIAI formed into rigid amyloid fibers agglutinating *S. mutans* when culturing biofilm. F: GQSIAI, SSHMCM and NQSVSI at 0.05 mg ml^−1^ did not affect planktonic *S. mutans* proliferation. Red arrow: rigid amyloid fibers aggregated by GQSIAI. **P < 0.01; *n* = 3.
