# Supplementary material for: The broad‐spectrum antibiofilm activity of amyloid‐forming hexapeptides
Source: Microb Biotechnol. 2020 Nov 28;14(2):656–67. doi: 10.1111/1751-7915.13721 (PMC7936291; doi:10.1111/1751-7915.13721)
Supplement: Supplementary file 1 — Fig. S1. Three‐dimensional reconstruction CLSM images of S. mutans biovolume and the calculated dead/live ratio of S. mutans cells. A to C: CLSM images showed that the biovolume of S. mutans cells were obviously decreased in AFhP(P3) group (B) when compared with control (A) and non‐AFhP(P8) group (C). D: No significant differences were found in the dead/live ratio of S. mutans cells among control, AFhPs (P1, P3, and P6) groups and non‐AFhPs groups (P5, P8 and P9). n = 3. Fig. S2. Cell cytotoxicity of AFhPs and non‐AFhPs. CCK‐8 assay showed that AFhP(P1) and AFhP(P3) at 0.05 mg ml−1 significantly decreased NOKs proliferation, while AFhP(P6) and non‐AFhPs (P5, P8 and P9) at 0.05 mg ml−1 had no significant influence on NOKs proliferation. *P < 0.05; n = 3. Fig. S3. AFhP(P3) at 0.05 mg ml−1 aggregated into long and thick rigid amyloid fibers, agglutinating S. mutans cells surface. Fig. S4. TEM images of AFhPs and non‐AFhPs on S. mutans biofilm formation. A and B: AFhP(P1) and AFhP(P6) at 0.05 mg ml−1 formed rigid amyloid fibers agglutinating S. mutans cells, respectively. C and D: Twisted amyloid fibers produced by S. mutans itself could be seen in non‐AFhP(P5) and non‐AFhP(P9) groups. Red arrow: rigid amyloid fibers aggregated by AFhPs; Blue arrow: twisted amyloid fibers produced by S. mutans. Fig. S5. Effect of AFhP and non‐AFhP on planktonic S.mutans. A: Naked eye view of the 24 h‐cultured planktonic S. mutans. There were more flocculent precipitates in 0.05 mg ml−1 AFhP(P3) treated group when comparing with control and 0.05 mg ml−1 non‐AFhP(P8) treated group. B: TEM results showed that AFhP(P3) aggregated into rigid amyloid fibers agglutinating S. mutans. Red arrow: rigid amyloid fibers aggregated by AFhP(P3). Fig. S6. Effect of AFhPs and non‐AFhPs on other planktonic microbes proliferation. A to D: Naked eye view of the 24 h‐cultured planktonic G+ bacteria (S. sanguis and S. aureus), G− bacteria (E. coli) and fungus (C. albicans) treated by 0.05 mg ml−1 hexapeptide [file MBT2-14-656-s001.docx]

**Appendix Figures and Figure legends：**

**
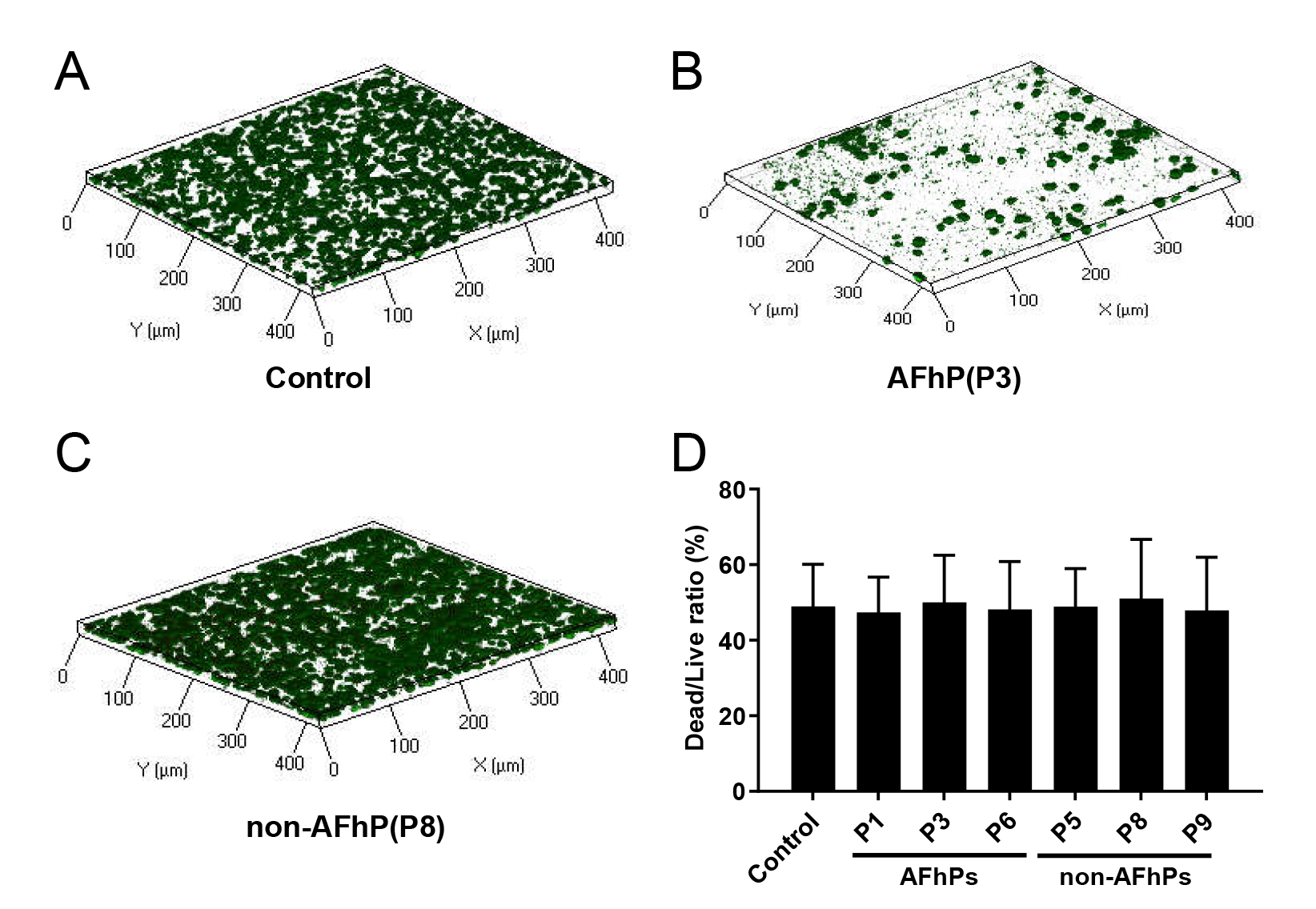
**

**Fig S1. Three-dimensional reconstruction CLSM images of *S. mutans* biovolume and the calculated dead/live ratio of *S. mutans* cells.** A to C: CLSM images showed that the biovolume of *S. mutans* cells were obviously decreased in AFhP(P3) group (B) when compared with control (A) and non-AFhP(P8) group (C). D: No significant differences were found in the dead/live ratio of *S. mutans* cells among control, AFhPs (P1, P3, and P6) groups and non-AFhPs groups (P5, P8 and P9). n=3.

**
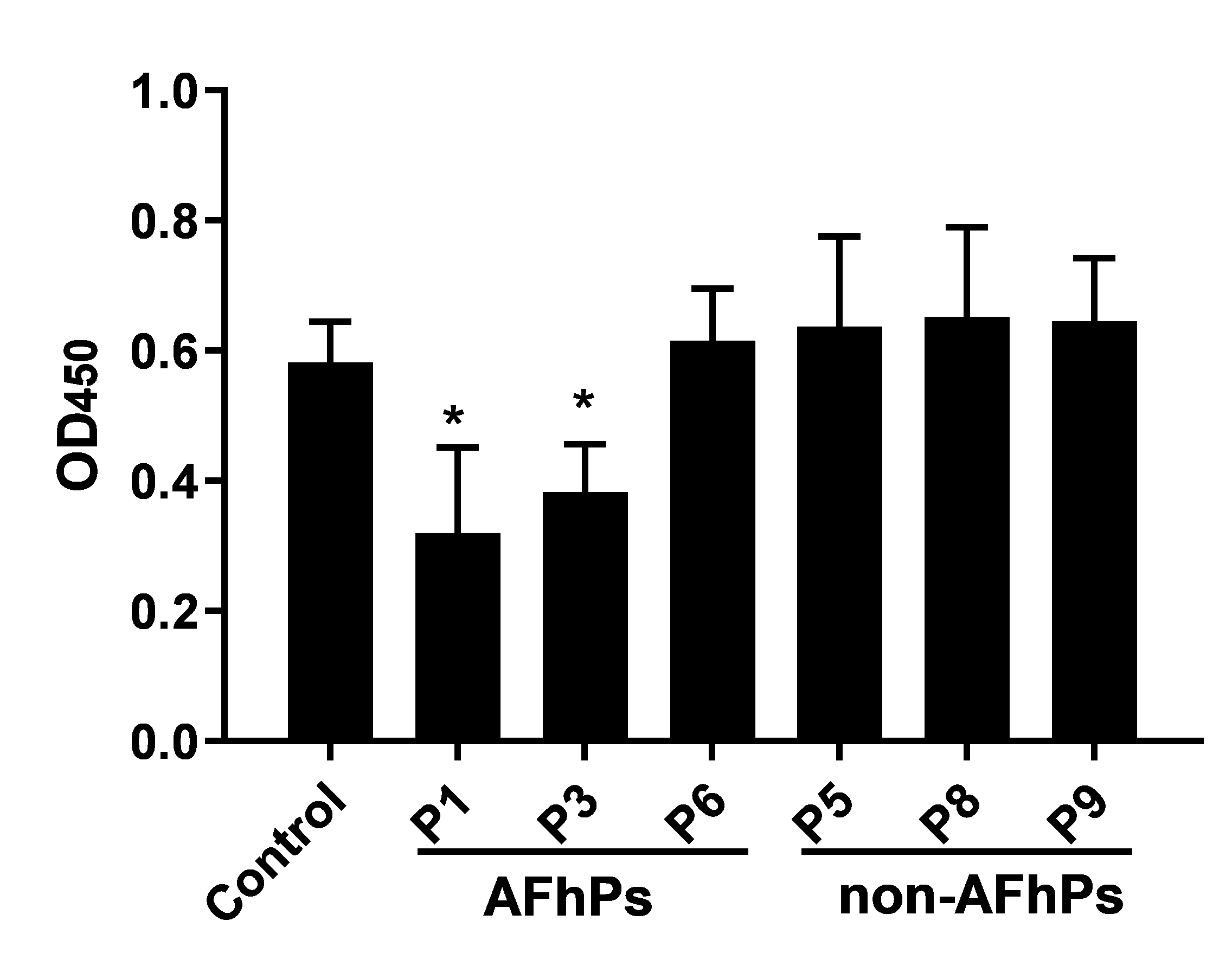
**

**Fig S2. Cell cytotoxicity of AFhPs and non-AFhPs.** CCK-8 assay showed that AFhP(P1) and AFhP(P3) at 0.05 mg/mL significantly decreased NOKs proliferation, while AFhP(P6) and non-AFhPs (P5, P8 and P9) at 0.05 mg/mL had no significant influence on NOKs proliferation. *: *p*<0.05; n=3.


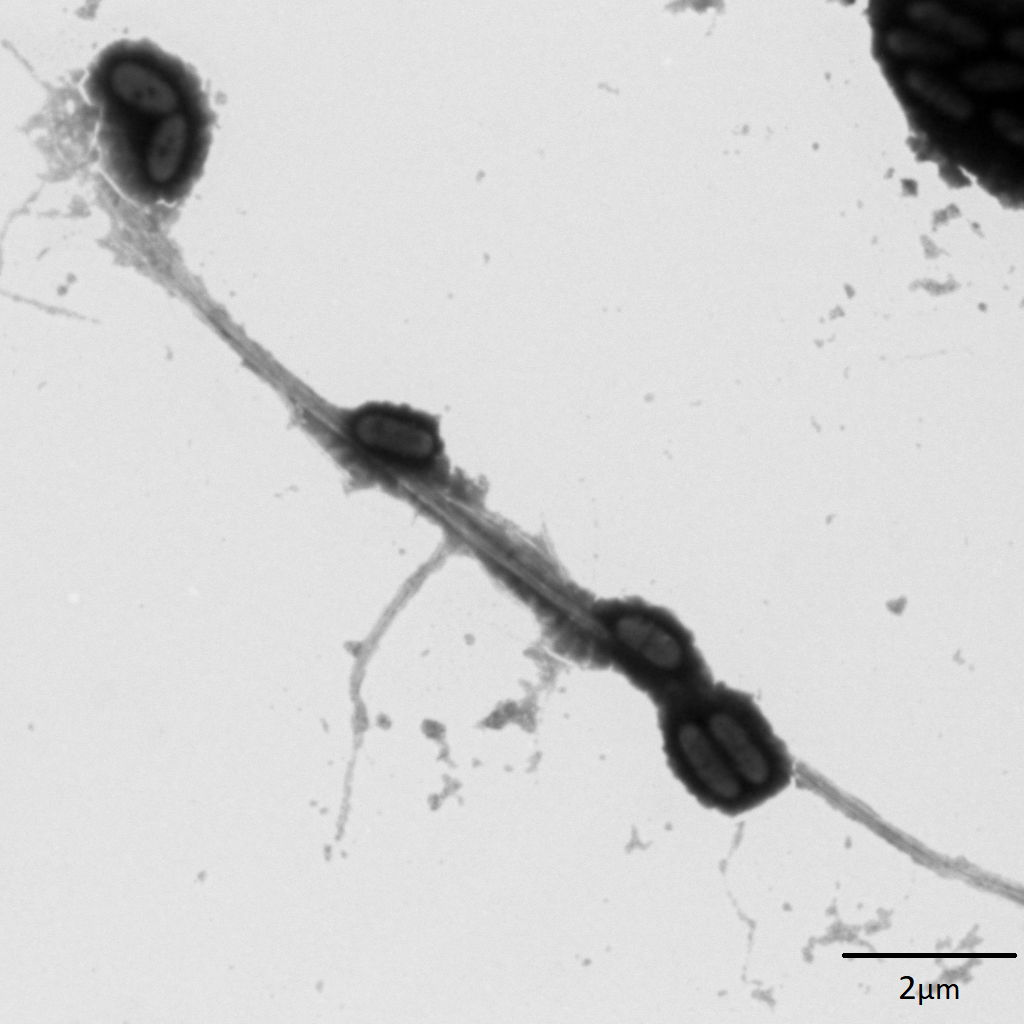


**Fig S3.** AFhP(P3) at 0.05 mg/mL aggregated into long and thick rigid amyloid fibers, agglutinating *S. mutans* cells surface.


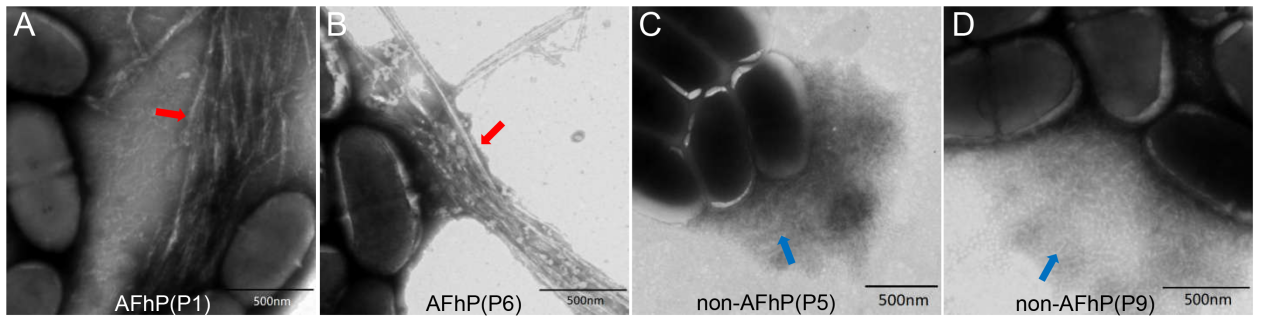


**Fig S4. TEM images of AFhPs and non-AFhPs on *S. mutans* biofilm formation.** A and B: AFhP(P1) and AFhP(P6) at 0.05 mg/mL formed rigid amyloid fibers agglutinating *S. mutans* cells, respectively. C and D: Twisted amyloid fibers produced by *S. mutans* itself could be seen in non-AFhP(P5) and non-AFhP(P9) groups. Red arrow: rigid amyloid fibers aggregated by AFhPs; Blue arrow: twisted amyloid fibers produced by *S. mutans*.


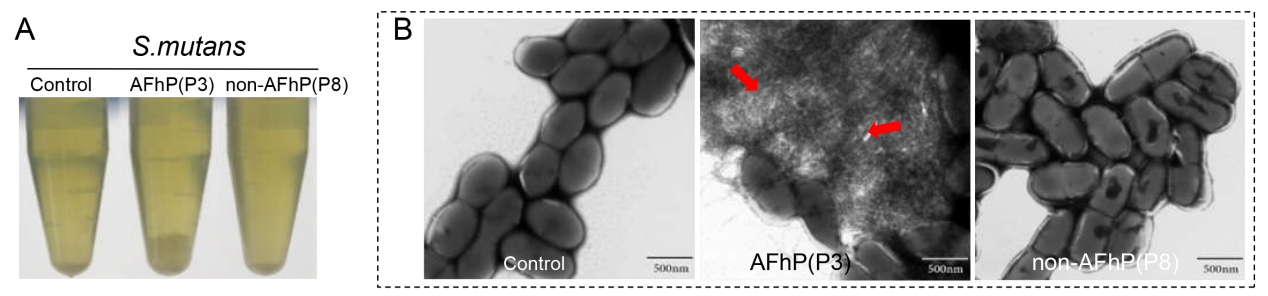


**Fig S5. Effect of AFhP and non-AFhP on planktonic *S.mutans.*** A: Naked eye view of the 24 h-cultured planktonic *S. mutans*. There were more flocculent precipitates in 0.05 mg/mL AFhP(P3) treated group when comparing with control and 0.05 mg/mL non-AFhP(P8) treated group. B: TEM results showed that AFhP(P3) aggregated into rigid amyloid fibers agglutinating *S.mutans*. Red arrow: rigid amyloid fibers aggregated by AFhP(P3).


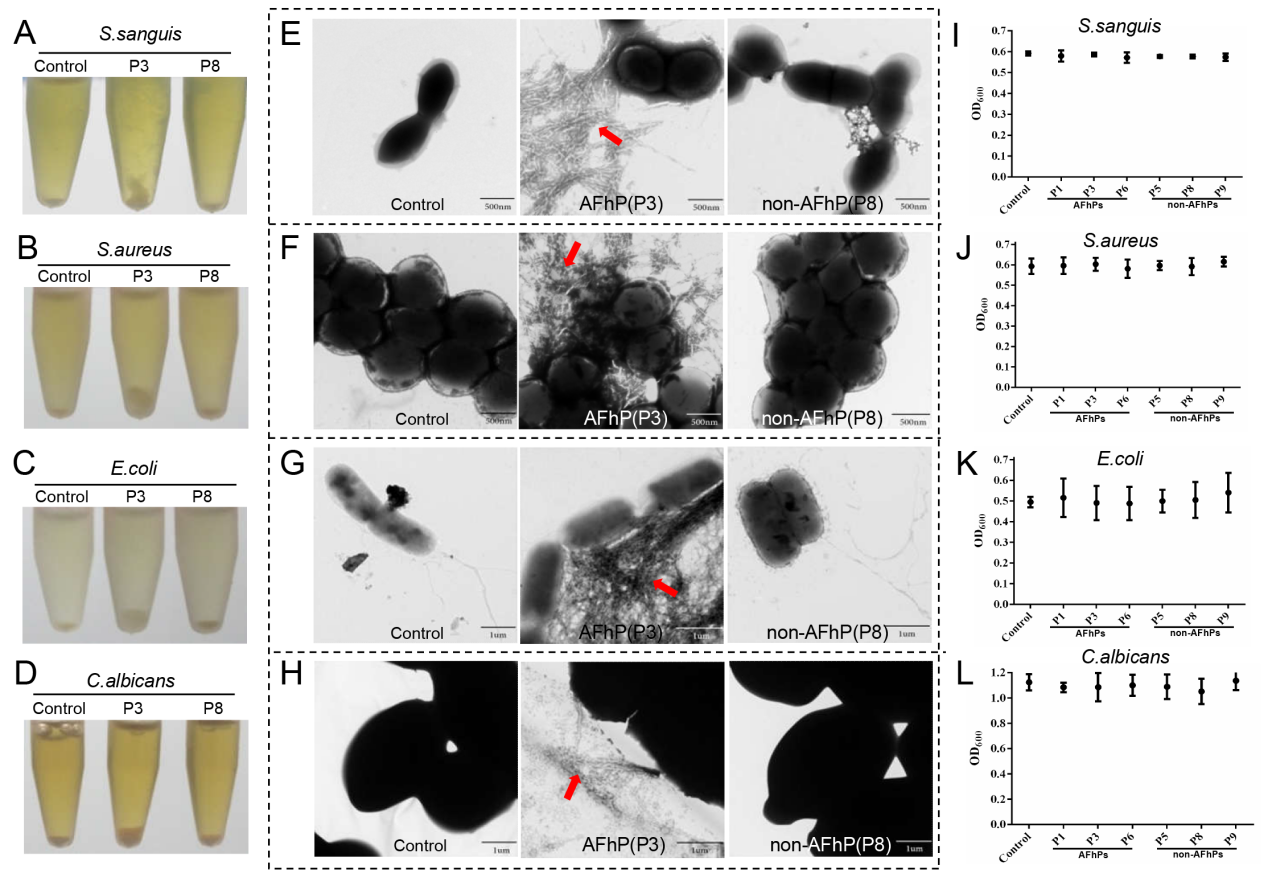


**Fig S6. Effect of AFhPs and non-AFhPs on other planktonic microbes proliferation.** A to D: Naked eye view of the 24 h-cultured planktonic G^+^ bacteria (*S. sanguis* and *S. aureus*), G^-^ bacteria (*E. coli*) and fungus (*C. albicans*) treated by 0.05 mg/mL hexapeptides or not. More flocculent precipitates were observed in AFhP(P3)-treated group. E to H: TEM results showed that 0.05 mg/mL AFhP(P3) aggregated into rigid amyloid fibers agglutinating *S. sanguis*, *S. aureus*, *E. coli* and *C. albicans*. I to L: AFhPs and non-AFhPs at a concentration of 0.05 mg/mL had no influence on the proliferation values of *S. sanguis,* *S. aureus*, *E. coli* and *C. albicans.* Red arrow: rigid amyloid fibers aggregated by AFhPs.


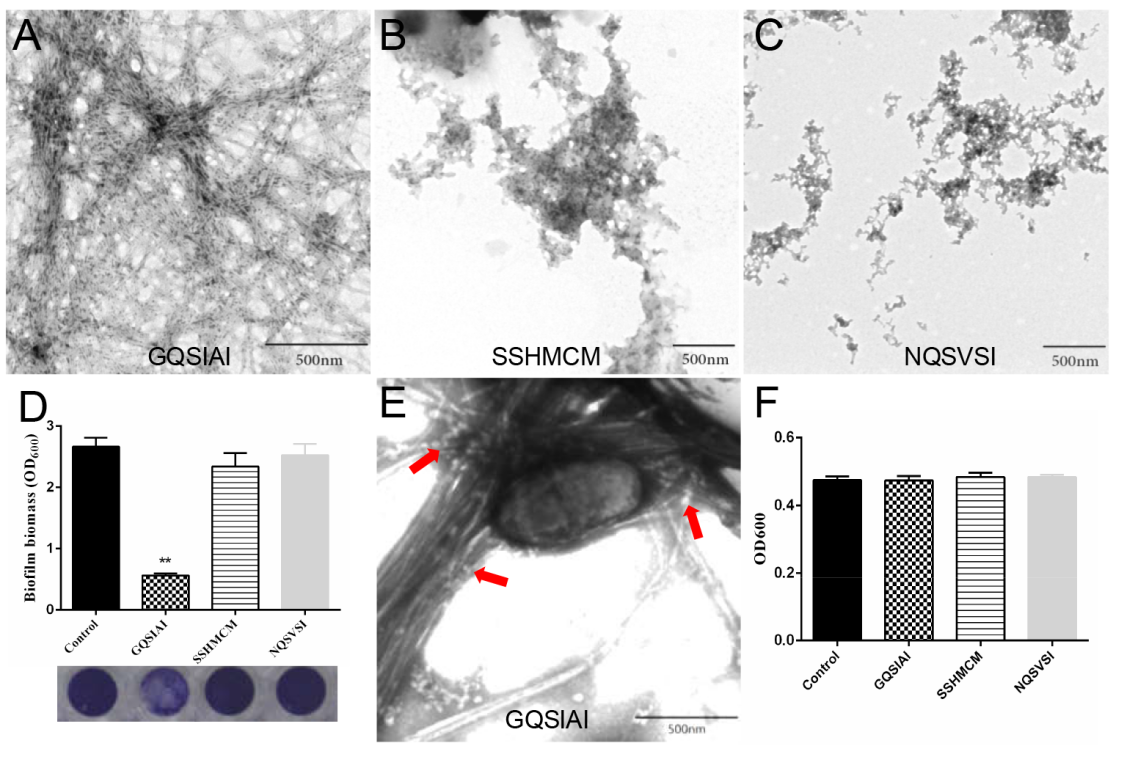


**Fig S7. Effect of hexapeptides derived from other species on *S.mutans* biofilm formation.** A to C: The aggregated status of 0.05 mg/mL GQSIAI (A), SSHMCM (B) and NQSVSI (C) observed by TEM. GQSIAI could aggregate into rigid amyloid fibers, while SSHMCM and NQSVSI could not. D: GQSIAI at 0.05 mg/mL significantly decreased *S. mutans* biofilm biomass, while SSHMCM and NQSVSI did not. E: GQSIAI formed into rigid amyloid fibers agglutinating *S. mutans* when culturing biofilm*.* F: GQSIAI, SSHMCM and NQSVSI at 0.05 mg/mL did not affect planktonic *S. mutans* proliferation. Red arrow: rigid amyloid fibers aggregated by GQSIAI. **: *p*<0.01; n=3.
